# Astrocytic Propagation of Tau in the Context of Alzheimer's Disease

**DOI:** 10.3389/fncel.2021.645233

**Published:** 2021-03-17

**Authors:** Rebecca M. Fleeman, Elizabeth A. Proctor

**Affiliations:** ^1^Department of Neurosurgery, Department of Pharmacology, College of Medicine, Pennsylvania State University (PSU), Hershey, PA, United States; ^2^Center for Neural Engineering, Pennsylvania State University (PSU), University Park, PA, United States; ^3^Department of Biomedical Engineering, Department of Engineering Science and Mechanics, Center for Neural Engineering, Pennsylvania State University (PSU), University Park, PA, United States

**Keywords:** Alzheimer's disease, tau propagation, astrocyte, inflammation, tau internalization

## Abstract

More than 6 million Americans are currently living with Alzheimer's disease (AD), and the incidence is growing rapidly with our aging population. Numerous therapeutics have failed to make it to the clinic, potentially due to a focus on presumptive pathogenic proteins instead of cell-type-specific signaling mechanisms. The tau propagation hypothesis that inter-neuronal tau transfer drives AD pathology has recently garnered attention, as accumulation of pathological tau in the brain has high clinical significance in correlating with progression of cognitive AD symptoms. However, studies on tau pathology in AD are classically neuron-centric and have greatly overlooked cell-type specific effects of tau internalization, degradation, and propagation. While the contribution of microglia to tau processing and propagation is beginning to be recognized and understood, astrocytes, glial cells in the brain important for maintaining neuronal metabolic, synaptic, trophic, and immune function which can produce, internalize, degrade, and propagate tau are understudied in their ability to affect AD progression through tau pathology. Here, we showcase evidence for whether tau uptake by astrocytes may be beneficial or detrimental to neuronal health and how astrocytes and their immunometabolic functions may be key targets for future successful AD therapies.

## Introduction

The prevalence of Alzheimer's disease (AD) is increasing rapidly in many countries due to an aging population and lack of successful therapeutics (Oh and Rabins, 2019). Unsuccessful therapeutic candidates may have failed for many reasons, including a strategy nearly exclusively focused on targeting the expression and aggregation of presumptive neurotoxic proteins at stages of disease when these processes may already be obsolete (Grubman et al., 2019; Mathys et al., 2019). These strategies have targeted what are known as the hallmarks of AD, amyloid-β (Aβ) plaques and hyperphosphorylated microtubule-associated protein tau (MAPT, or tau) aggregates (Franco and Cedazo-Minguez, 2014; Tai et al., 2015; Elmaleh et al., 2019; Tzioras et al., 2019), but a mechanistic understanding of how the progression of these pathologies leads to neuron death and cognitive decline is lacking. Accumulation of pathological tau in the brain is clinically significant as it correlates with progression of cognitive symptoms better than other markers, including Aβ (Arriagada et al., 1992; Brier et al., 2016). Tau is a cytoskeletal protein that stabilizes microtubules of neuron axons (Yamada, 2017). When monomeric tau becomes hyperphosphorylated (Ksiezak-Reding et al., 1992), it can disassociate from the microtubule and aggregate into pathological oligomers (Mandelkow and Mandelkow, 2012; Fichou et al., 2019). Pathological tau aggregates can develop into neurofibrillary tangles inside the neuron, leading to neuronal degeneration by unknown mechanisms (Mandelkow and Mandelkow, 2012; Fichou et al., 2019). Initiation of tau hyperphosphorylation is not well-understood but defining the molecular mechanisms of initial hyperphosphorylation induction and propagation of tauopathy through the brain is paramount to slowing or stopping the spread of pathological tau and identifying new candidates for effective AD therapeutics.

Neurons can secrete tau into the extracellular space, as evidenced by quantifiable release from stimulated neurons *in vitro* and *in vivo* (Pooler et al., 2013; Wu et al., 2016; Pernègre et al., 2019). This finding has influenced the tau propagation hypothesis, which proposes that inter-neuronal tau transfer drives AD pathology (Takeda, 2019). Importantly, tau is also well-known to spread through synaptic connections (Spires-Jones and Hyman, 2014). Tauopathy is characteristically associated with neurons because neurons express far more tau than other cell types in the brain (Mandelkow and Mandelkow, 2012; Guo et al., 2017). However, tau internalization has been observed in astrocytes and microglia, both *in vivo* and *in vitro* (Ikeda et al., 2005; de Calignon et al., 2012; Asai et al., 2015; Bolós et al., 2016), with little known about the effects of tau in glial cells. Aging-related tau astrogliopathy (ARTAG) is the umbrella term used to define astrocytes with abnormal accumulation of pathological tau. ARTAG is found in not only AD, but also neurodegenerative diseases such as Parkinson's disease and chronic traumatic encephalopathy (Ferrer et al., 2018; Kovacs, 2020). Thorn-shape astrocytes (TSA) and granular-fuzzy astrocytes (GFA) are the astrocytic morphologies characteristic of ARTAG. In particular, astrocytes can both degrade and propagate tau (Martini-Stoica et al., 2018), and thus warrant a closer look in terms of propagation of AD pathology. Astrocytes are a particularly interesting target due to their role in the tripartite synapse (Martini-Stoica et al., 2018), optimally positioning them to receive tau released by neurons. Astrocytes also regulate neuroinflammatory responses through cytokine signaling (Sofroniew and Vinters, 2010; Phillips et al., 2014; Heneka et al., 2015; Wood et al., 2015; Leyns and Holtzman, 2017). Indeed, AD patients are known to have high levels of neuroinflammation (Heneka et al., 2015), with some studies supporting neuroinflammation as a key driver of AD pathology (Heneka et al., 2015; Ising et al., 2019). Tau accumulation is promoted in the presence of pro-inflammatory cytokines such as TNFα (Gorlovoy et al., 2009), and internalization of pathological tau may in turn alter astrocytic cytokine secretion, suggesting a positive feedback mechanism by which tau and inflammation may drive AD pathology. Sustained astrogliosis [astrocytic activation (Sofroniew and Vinters, 2010)] can diminish the metabolic, trophic, synaptic, and immune support that astrocytes provide to neurons, and is detrimental to neuronal health (Phillips et al., 2014; Heneka et al., 2015; Verkhratsky et al., 2015). The mechanism by which astrocytic tau internalization may promote tau propagation, astrogliosis, inflammatory cytokine secretion, and subsequent neurodegeneration is unknown (Gomez-Arboledas et al., 2018; Martini-Stoica et al., 2018; Takeda, 2019). Defining the molecular underpinnings of this relationship will aid in developing a mechanistic roadmap of AD progression, from the initial insult of astrocytic tau internalization to the end result of progressive neuronal death. This review focuses on the role of astrocytes in tau propagation, and how astrocytic internalization and failure to degrade tau can promote the spread of disease pathology. We explore previous findings that suggest how tau uptake by astrocytes may be beneficial or detrimental to neuronal health and consider how the repercussions of astrocytic tau propagation fit into the larger picture of neurodegeneration.

## Tau Internalization In Astrocytes

Healthy neurons express robust levels of tau that localize to axonal microtubules for transport and stabilization (Kahlson and Colodner, 2015). In neurons of AD patients, tau becomes hyperphosphorylated, causing it to disassociate from the microtubule and form pathological aggregates that spread in a characteristic pattern, originating in the entorhinal cortex and locus coeruleus and progressing to the neocortex (Grundke-Iqbal et al., 1986; Braak and Braak, 1991; Hyman, 1997; Franzmeier et al., 2020). Healthy astrocytes express very low levels of tau (Müller et al., 1997; Kahlson and Colodner, 2015; Perea et al., 2019). Despite low endogenous astrocytic expression, hyperphosphorylated tau has been observed in astrocytes in AD (Probst et al., 1982; Kahlson and Colodner, 2015; Ferrer et al., 2018), leading us to question the source of this tau if it does not originate in the astrocyte. Primary human astrocytes do not increase expression of tau when stimulated with Aβ (Chiarini et al., 2017), suggesting the observed increase in astrocytic tau in AD is due to internalization of exogenous tau. Mechanisms of tau secretion and internalization by neurons are well-understood (Holmes et al., 2013; Pooler et al., 2013; Yamada, 2017; Pernègre et al., 2019; Rauch et al., 2020), but transmission of tau between astrocytes and the pathological consequences of astrocytic tau uptake remain incompletely described (Kahlson and Colodner, 2015; Yamada, 2017; Perea et al., 2019). Tau can be internalized by astrocytes as monomers (Perea et al., 2018), truncated preformed tau fibrils (PFF) (Martini-Stoica et al., 2018), or aggregates (de Calignon et al., 2012; Asai et al., 2015). Astrocytes internalize tau monomers *in vitro* (Perea et al., 2019), and *ex vivo* experiments have demonstrated that astrocytes take up hyperphosphorylated tau from degenerating and injured neurons (de Calignon et al., 2012). When neurons accumulate neurofibrillary tau tangles they die and leave “ghost tangles,” stable tau aggregates without the presence of a cell nucleus, in the extracellular space (Gómez-Ramos et al., 2009). Astrocytes can infiltrate ghost tangles with their processes, contributing to tau accumulation in astrocytes in AD (Probst et al., 1982; Irwin et al., 2012; Perez-Nievas and Serrano-Pozo, 2018). Cultured human astrocytes internalize and release tau at a steady rate, even without stimulation (Chiarini et al., 2017). Further observations indicate that tau monomers are internalized by astrocytes through a non-heparan sulfate proteoglycan (HSPG)-mediated mechanism (Perea et al., 2018) and PFFs are internalized by astrocytes *via* phagocytosis (Martini-Stoica et al., 2018). Additionally, tau internalization by astrocytes can come from phagocytosis of plaque-associated dystrophic neurites (Gomez-Arboledas et al., 2018) and synapses containing tau aggregates (Sanchez-Mico et al., 2020). Astrocytes are well-known to react against Aβ plaques (Gomez-Arboledas et al., 2018), and astrogliosis stemming from Aβ pathology can stimulate greater phagocytic behavior in astrocytes, potentially increasing tau internalization (Wang and Ye, 2021). Further studies are required to determine whether neuronal receptors of tau [including LRP1 (Rauch et al., 2020) and HSPGs (Holmes et al., 2013)] are involved in astrocytic tau internalization or if novel mechanisms are at work.

## Factors Influencing Astrocytic Tau Internalization

A number of AD-relevant factors may alter levels of astrocytic tau internalization. Elevated cholesterol is known to promote tau phosphorylation and subsequent tauopathy (Distl et al., 2001; Rahman et al., 2005; Ohm and Meske, 2006), suggesting that cholesterol dysregulation may be a key factor increasing tau propagation by astrocytes. Astrocytes are the primary producers of apolipoprotein E (APOE) in the brain, and APOE carries cholesterol from astrocytes to neurons (Mahley, 2016a). Of the 3 APOE isoforms, APOE ε4 is the strongest and most common genetic risk factor for late-onset AD as compared to the most common APOE isoform, APOE ε3, while the third isoform, APOE ε2, may increase risk for primary tauopathies (Corder et al., 1993; Jones and Rebeck, 2018; Zhao et al., 2018). APOE ε4 increases the risk of AD by 4- to 14-fold (Liu et al., 2017) and decreases age of disease onset by 8–12 years (Mahley, 2016a; Belloy et al., 2019). APOE ε4 carriers exhibit increased astrocytic cholesterol biosynthesis and intracellular cholesterol accumulation (Heeren et al., 2004; Chen et al., 2014; Mahley, 2016b; Lin et al., 2018). This impaired cholesterol trafficking leads to endosomal dysfunction, resulting in AD pathology (Chen et al., 2014). Despite the stark increase in AD risk conveyed by APOE ε4, its mechanism, and especially its impact on development and progression of tau pathology, remains incompletely understood (Williams et al., 2020). Elevated levels of cholesterol observed in neural cells of APOE ε4 carriers (Heeren et al., 2004; Chen et al., 2014; Mahley, 2016b; Lin et al., 2018), in tandem with increased tauopathy due to defects in cholesterol metabolism (Distl et al., 2001; Rahman et al., 2005), suggest heightened cholesterol levels may promote tau propagation. Increased cholesterol levels also increase phagocytic behavior in macrophages (Ares et al., 2019) and macrophage-like cells (Bryan et al., 2014). Thus, APOE ε4 astrocytes, which have increased cholesterol levels (Lin et al., 2018), may internalize greater amounts of tau and lead to accelerated disease progression if internalization triggers an inflammatory cascade. On the other hand, a study of *post-mortem* human brain tissue found no effect of APOE genotype on ARTAG (Lace et al., 2012; Kovacs et al., 2017). Previous studies indicate decreases in APOE ε4 astrocyte autophagy with Aβ internalization (Simonovitch et al., 2016), yet no such study has been carried out for tau internalization. Finally, APOE ε2 has been shown to increase primary tauopathy pathology when Aβ is not present (Zhao et al., 2018) and thus, effects of APOE may be dependent on the presence of Aβ pathology. Aβ is known to increase the total tau accumulation in astrocytes (Richetin et al., 2020), thus future studies are needed to parse out the effects of APOE genotype and Aβ on astrocytic tau accumulation.

Another factor influencing astrocytic tau internalization and subsequent propagation is extracellular tau concentration. Astrocytic tau internalization has been observed *in vitro* upon addition of varying concentrations of tau (Asai et al., 2015; Piacentini et al., 2017; Martini-Stoica et al., 2018; Perea et al., 2019). While the intraneuronal physiological concentration of tau is ~2 μM (Avila, 2010), extracellular tau concentrations in the cerebrospinal fluid (CSF) are 279–596 nM in AD patients and 29–134 nM in cognitively normal, age-matched controls (Tato et al., 1995; Han et al., 2017). Therefore, the 2 μM used in tau internalization studies is ~5-fold greater than what is seen in CSF of AD patients and may lead to misleading results in the case of non-specific internalization, like pinocytosis. Additionally, the aggregation state of tau (oligomer, fibril, monomer, etc), as well as the isoform (0N3R, 0N4R, 1N3R, 1N4R, 2N3R, and 2N4R) may modulate the likelihood and rate of internalization (Fichou et al., 2019). All six tau isoforms are expressed in AD, and tau tangles in *post mortem* AD brains have an even ratio of 3R to 4R isoforms (Guo et al., 2017; Pernègre et al., 2019; Zhang et al., 2019). While 3R and 4R tau are both found in neuronal tau pathology in the AD brain, studies describing ARTAG in AD report mainly 4R tau pathology in astrocytes (Ferrer et al., 2014; Kovacs et al., 2016; Kovacs, 2020). Most internalization studies use only 2N4R tau (Pooler et al., 2012; Holmes et al., 2013; Asai et al., 2015; Wang et al., 2017; Perea et al., 2019), which is disadvantageous because 1N isoforms are the most common in adult human brain and CSF (Barthélemy et al., 2016; Richetin et al., 2020). A recent study found 3R astrocytic tau inclusions in the hilus of the hippocampus associated with increased AD Braak stage and correlating with synaptic protein levels (Richetin et al., 2020), suggesting a relationship between astrocytic tau accumulation and synaptic degradation in AD. Microglia internalize more 2N4R tau aggregates *in vitro* than do astrocytes or neurons (Asai et al., 2015), and thus microglia have come under increased scrutiny as the main cell type responsible for tau propagation. However, astrocytes play different support roles than microglia, namely in metabolic, trophic, and synaptic functions, which could be detrimentally influenced more strongly by smaller amounts of tau internalization than those affecting microglia. A comparison of astrocytic internalization in both isolated and multi-cell type systems is crucial to fully understand this complex relationship.

## Astrocytic Degradation and Propagation of Internalized Tau

Similarly to the mechanism and cellular consequences of astrocytic tau internalization, the fate of internalized tau has been understudied (Kahlson and Colodner, 2015; Martini-Stoica et al., 2018). Astrocytic tau accumulation is seen in primary tauopathies such as supranuclear palsy (PSP) and corticobasal degeneration (CBD) (Leyns and Holtzman, 2017). In these diseases, as well as in secondary tauopathies such as AD, ARTAG with perinuclear hyperphosphorylated tau aggregates have been observed (Kahlson and Colodner, 2015; Leyns and Holtzman, 2017). Tau accumulation in astrocytes has been demonstrated robustly in AD *post mortem* brains (Leyns and Holtzman, 2017), yet the mechanism of tau transport into and out of astrocytes in AD remains unknown (Perez-Nievas and Serrano-Pozo, 2018).

**Figure 1:**
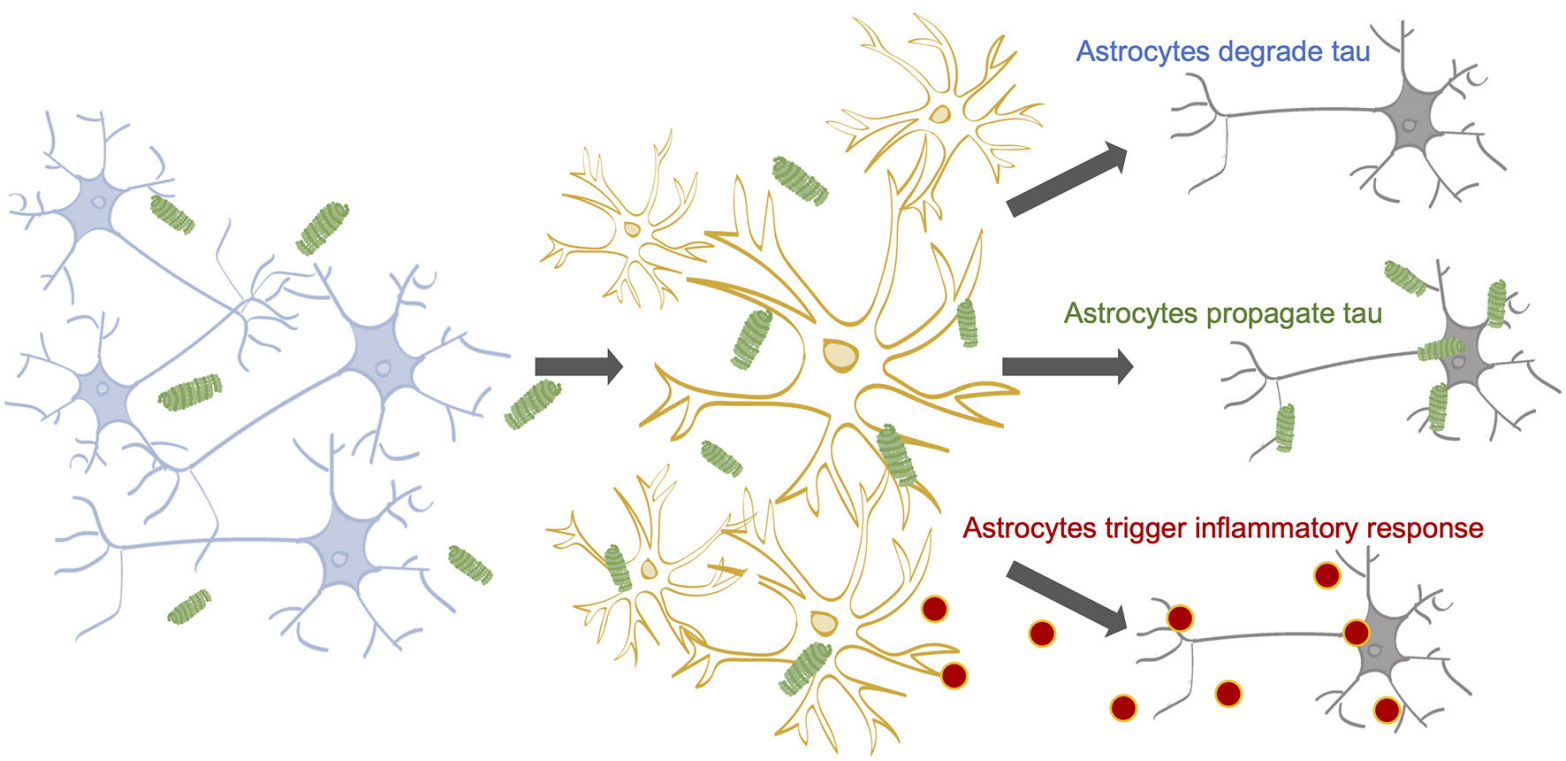
The impact of astrocytes on tau propagation in AD. Neurons contain tau on their microtubules (Yamada, 2017). Tau can become hyperphosphorylated and dissociate from the microtubules due to a myriad of factors (Mandelkow and Mandelkow, 2012; Fichou et al., 2019). Stimulated or dying neurons can release tau into the extracellular space (Pooler et al., 2013; Wu et al., 2016; Pernègre et al., 2019) 14-16, where astrocytes are known to internalize tau (de Calignon et al., 2012; Asai et al., 2015; Martini-Stoica et al., 2018; Perea et al., 2018, 2019). Astrocytic internalization rates may be affected by a number of factors, including cholesterol levels; APOE status; and tau concentration, aggregation state, and isoform. Following internalization, astrocytes may (1) degrade the internalized tau, (2) release the tau back out of the cell, potentially propagating the pathological tau to healthy neurons, and (3) accumulate tau, potentially triggering inflammatory cytokine release, which can harm neuronal health.

Once internalized by astrocytes, three outcomes for tau are possible: degradation, re-release, or accumulation (Figure 1). Astrocytes may act as a specialized tau vacuum, engulfing pathological tau from the extracellular matrix of the brain and breaking it down to be recycled. Pre-formed tau fibrils, internalized by astrocytes through micropinocytosis, are degraded by lysosomes *in vitro* (Martini-Stoica et al., 2018). When the lysosomal Transcription Factor EB (TFEB) was overexpressed in astrocytes *in vitro* and *in vivo*, both incidence and spread of pathological tau was decreased, indicating that astrocytes have the ability to both degrade and propagate tau (Martini-Stoica et al., 2018). Additionally, tau is an intrinsically disordered protein, increasing its likelihood for degradation (Ferrari and Rüdiger, 2018; Ukmar-Godec et al., 2020). However, it was recently shown that the hyperphosphorylation and aggregation of tau decreases its proteasomal degradation, which may lead to further tau accumulation (Ukmar-Godec et al., 2020). These findings suggest astrocytic tau internalization as a potential therapeutic target for tauopathy, where increasing tau internalization could prevent tau propagation.

Discrepancies in the literature over tau expression and secretion in astrocytes warrant greater scrutiny to truly understand cell-type specific changes in the AD and aging brain. Contradicting results have been found between murine and human systems, and between *in vitro* and *ex vivo* paradigms. Each of these experimental systems has caveats, and thus identifying which results are most relevant to disease in patients is difficult. Primary human astrocytes display diffuse tau expression and secrete tau in exosomes (Chiarini et al., 2017). Conversely, while primary murine microglia take up 2N4R tau oligomers by phagocytosis and release tau in exosomes (Asai et al., 2015), no tau has been found in murine astrocytic exosomes (Asai et al., 2015). However, exosomal tau is not the majority of tau in patient CSF, and therefore glial exosomal tau may not be a disease-relevant pathology (Yamada, 2017). When AD-pathological human tau was injected into wild-type mice, only neurons propagated the injected tau (Narasimhan et al., 2017), which contradicts studies that report tau inclusions in astrocytes of AD patients *post mortem* (Kovacs et al., 2016; Leyns and Holtzman, 2017). These conflicting findings underscore that astrocytic tau uptake and propagation remains poorly studied in the AD field, and thus the role that astrocytes play in tau propagation in AD remains unknown.

## Downstream Effects of Astrocytic Tau Internalization

The majority of AD tau pathology research focuses on neurons, yet astrocytes play a vital role in maintaining neuronal fitness in the presence of AD pathology (Leyns and Holtzman, 2017). As part of the tripartite synapse, astrocytes are well-positioned to receive tau released from neurons and internalize extracellular tau (Martini-Stoica et al., 2018). Tau that remains in the extracellular space has been well-established to contribute to the propagation of tau pathology (Wu et al., 2016; Yamada, 2017), thus removal by internalization is an important mechanism by which astrocytes can aid in maintaining brain health. However, there is little understanding of the repercussions of astrocytic tau internalization for disease progression and outcome. While uptake of tau from the extracellular space may aid in decreasing direct neurotoxic effects of tau on neurons, this uptake and presence of pathological tau species intracellularly could have detrimental effects on astrocytes themselves. Astrocytic support functions to neurons could thus be negatively impacted (Kovacs, 2020), indirectly decreasing neuronal health and resilience to insult.

Tau is normally expressed at very low levels in astrocytes (Müller et al., 1997; Kahlson and Colodner, 2015; Perea et al., 2019). When high levels of tau have been artificially induced in astrocytes, a body of evidence suggests it is detrimental to cellular function and viability (Yoshiyama et al., 2003; Dabir et al., 2006; Prebil et al., 2011; Allen, 2014). Transgenic mice expressing wild-type and mutant human tau protein in astrocytes have decreased levels of glutamate transporters in the brain and spinal cord (Dabir et al., 2006) as compared to non-transgenic counterparts. Decreased glutamate transporter expression can impair neuronal signaling and cause glutamate toxicity, and inducing human tau expression in primary rat astrocytes causes cytoskeletal collapse followed by astrocyte death (Yoshiyama et al., 2003). Decreased astrocytic viability also decreases critical neuronal metabolic support, resulting in greater potential for neuron death (Prebil et al., 2011; Allen, 2014). Recent evidence also suggests that synaptic density is altered in a 1N3R tau overexpression system in astrocytes (Richetin et al., 2020) and that 1N3R overexpression also impairs mitochondrial transport and calcium homeostasis. Additionally, astrocytes overexpressing 1N3R tau in the hilus impair inhibitory neurons and cause asynchronous activity in neurons, leading to decreased spatial memory in mice (Richetin et al., 2020). Taken together, this evidence and the innate phagocytic properties of astrocytes (Allen, 2014) underlines the importance of studying astrocytes in terms of tau propagation and as a potential additional driver of tau-related neurodegeneration in AD.

In addition to metabolic and neurotransmitter dysregulation caused by astrocytes, chronic exposure to inflammatory cytokines is well-known to decrease neuronal health (Kennedy and Silver, 2015; Leyns and Holtzman, 2017). Glial-expressed cytokines such as IL-1β, TNFα, and IL-6 are upregulated in AD (Fillit et al., 1991; Blum-Degena et al., 1995; Benzing et al., 1999; Kálmán et al., 2009), and chronic activation of glial cells has been postulated to increase tau pathology (Leyns and Holtzman, 2017; Ising et al., 2019). Astrocytes are a key player in regulating cytokine secretion in the brain, an important task for both maintenance signaling and injury communication between cells. Astrocytic tau sequestration may elicit an inflammatory response from astrocytes, and in turn, these pro-inflammatory signals can decrease neuronal health (Han et al., 2017; Martini-Stoica et al., 2018). While there is no current evidence for how stimulation of astrocytes with tau affects cytokine secretion, primary human astrocytes stimulated with TNFα or IL-1β produce more inflammatory cytokines including IL-1β, TNFα, IL-6, IL-1ra, MIP-1α (CCL3), and RANTES (CCL5) (Choi et al., 2014). Additionally, cultured neurons exposed to inflammatory cytokine TNFα have increased tau pathology (Gorlovoy et al., 2009), demonstrating the potential of inflammatory cascades to affect tau pathology indirectly and also cyclically, with inflammation increasing tau accumulation and tau accumulation increasing inflammation. *Ex vivo*, transgenic mutant tau mice exhibit glial activation, with increased release of pro-inflammatory cytokines (Wang et al., 2017; Zhang et al., 2019), yet no direct study of effects of tau on astrocytes in cell culture have been performed. It is notable that while a number of studies have examined diseased tissue and shown correlation between tau and inflammation, no studies exist which test the causative mechanism of the effects of astrocytic tau internalization on both astrocytes and neurons. Determining precise cytokine reactions caused by astrocytic tau internalization could help determine the character and severity of the impact astrocytic tau internalization has on propagating tau pathology.

Finally, tau internalization by astrocytes may lead to dysfunction of the blood-brain barrier (BBB) through two key avenues: increased astrogliosis and decreased levels of tight junction and adhesion proteins. The BBB is a critical structure for protecting the brain microenvironment from toxins, as well as an important passageway for vital nutrients to enter the tissue. Astrocytes are an essential component of the BBB's multicellular selective barrier between peripheral blood flow and the brain parenchyma, with their endfeet forming a sheath around the vessels that produce the basement membrane for the parenchyma (Daneman and Prat, 2015). Damage to the BBB is well-documented in AD; it is less appreciated that BBB damage is also present in primary tauopathies, suggesting aberrant tau as a key contributor to BBB damage in AD (Blair et al., 2015; Majerova et al., 2019). Notably, ARTAG commonly localizes to perivascular astrocytes (Forman et al., 2005; Okamoto et al., 2019), further bolstering the connection between astrocytic tau pathology and BBB damage. Overexpression of human tau is known to disrupt astrocytic BBB functions (Kahlson and Colodner, 2015). *Ex vivo* studies of transgenic mice expressing mutant human tau demonstrate that phosphorylated tau from neurons of the entorhinal cortex is internalized by astrocytes, causing an increase in GFAP expression when compared to wild-type mice, indicative of astrogliosis (de Calignon et al., 2012). Additional studies have confirmed that transgenic mice expressing mutant tau exhibit significant astrogliosis that increases with age (Wes et al., 2014; Blair et al., 2015). Reactive astrogliosis induces inflammatory cytokine production, where cytokines such as VEGF-A can decrease levels of tight junction proteins vital to BBB function (Daneman and Prat, 2015). Notably, the onset of neuroinflammation precedes BBB dysfunction in transgenic mutant tau mice (Blair et al., 2015), suggesting astrocytes secrete inflammatory cytokines damaging to the BBB. Recent studies have begun to identify the effects of pathological tau on endothelial cells of the BBB, showing that pathological tau deteriorates brain microvasculature and increases senesce genes, leading to impaired blood flow (Bennett et al., 2018; Bryant et al., 2020). However, because the majority of studies of tau pathology in AD focus on neurons, the mechanism of BBB damage due to astrocytic tau internalization remains only indirectly studied. Deciphering the potential mechanisms by which astrocytic tau internalization may affect BBB integrity is critical for understanding neurodegenerative pathology and developing novel therapeutic strategies.

As we have presented, the phagocytic behavior of astrocytes removes toxic tau from the extracellular space (Kovacs, 2020), but negative consequences can occur if internalization is not immediately followed by degradation. Rapid removal of tau by cells other than neurons [e.g., microglia] can thus also contribute to neuronal health (Luo et al., 2015; Bolós et al., 2016). However, microglia are likely to participate in tau seeding and propagation more often than in mitigation (Asai et al., 2015; Perea et al., 2018). While astrocytic internalization holds promise as a potential target for modulating the toxicity of pathological tau species, both potential benefits and possible indirect negative effects on neuronal health need to be weighed before this phenomenon can be considered for developing therapeutic strategies against neurodegeneration. If astrocytes internalize tau and the majority is degraded, successful therapeutics may be found in enhancement of the yet-uncharacterized astrocytic internalization mechanism of various tau isoforms. Conversely, if future studies find that astrocytes internalize pathological tau only to spread it to nearby healthy neurons, inhibition of the secretion mechanism (also yet to be characterized) could be targeted to stop the spread of tau pathology in the AD brain. Finally, if the disease-relevant insult due to astrocytic tau internalization is secondary to the spread of tau pathology, such as BBB damage, pro-inflammatory cytokine cascades, or metabolic dysfunction, such evidence would further support the vascular, inflammatory, and metabolic hypotheses of AD, respectively, and further investigation of these downstream effects could serve to identify targets in those domains.

## Conclusion

The majority of tau pathology research in the field of AD focuses on neurons. Recently, studies have begun to shift toward elucidating the effects of pathological tau on glial cell types (Liddelow and Sofroniew, 2019; Kovacs, 2020), but the role of astrocytes in propagating tauopathy is still vastly underappreciated, even though astrocytes play a vital role in maintaining neuronal fitness. Evidence suggests that astrocytic tau internalization may be the lynchpin of several disparate hypotheses of AD, including the proteinopathy, inflammatory, vascular, and metabolic hypotheses. As part of the tripartite synapse, astrocytes are well-positioned to receive tau released from neurons and to internalize extracellular tau (Martini-Stoica et al., 2018). Tau released by neurons into the extracellular space contributes to tau propagation (Wu et al., 2016; Yamada, 2017), thus the ability of astrocytes to internalize extracellular tau could serve to halt the progression of tauopathy. Here, we present evidence for dual, opposing roles of astrocytic tau sequestration: as either a reactionary mechanism for clearance and degradation of tau from the extracellular space, or as a temporary holding place before re-release and propagation of pathological tau to more neurons. While contained within astrocytes, internalized tau may initialize cascades leading to astrogliosis, producing inflammatory responses as secreted cytokines, and thereby decrease neuronal health. Astrocytes may also internalize tau and fail to degrade it, leading to propagation of pathological tau out to more neurons. Astrocytes are largely stationary, but methods of release such as exocytosis, transduction through plasma membrane channels and transporters, and exosome release may give astrocytes the ability to propagate pathological tau to nearby neurons. Elucidation of the mechanism of astrocytic involvement in tau propagation could hold the key to uncovering novel therapeutic directions (Table 1). Without addressing the critical gaps in knowledge connecting astrocytic behavior with tau degradation and propagation tauopathy in AD, we are missing key opportunities to diversify our approaches to someday prevent, halt, and even reverse neurodegeneration.

**Table 1:** Priorities for future directions in astrocytic tau propagation in the context of AD.

**Known**	**Unknown**
Astrocytes have very low endogenous levels of tau but can internalize tau from the extracellular matrix and ghost tangles.	What is the mechanism for monomeric tau internalization in astrocytes?
Astrocytes play a key role in neuronal health and astrocytic tau internalization alters astrocyte morphology and function.	At what rate do astrocytes degrade/propagate tau?
While microglia also play a role in tau internalization, astrocytic changes are understudied in the context of tau pathology in AD.	What are the repercussions to neurons when astrocytes internalize tau?

## Author Contributions

RF and EP wrote the article.

## Conflict of Interest

The authors declare that the research was conducted in the absence of any commercial or financial relationships that could be construed as a potential conflict of interest.
